# Retrospect

**Published:** 1887-12-31

**Authors:** 


### RETROSPECT,

Look closely at the Might-have-beens of fate,
The gravestones raised o'er dead and buried hope,
And say, couldst thou recast thy horoscope,
And bring thy dead to life, wouldst thou debate
If it were wisdom to unlock the gate
God shut upon the paths thou fain hadst trod,
Or dig from under Time's green swathing sod,
Things once beloved, thou now perchance wouldst hate?

Not so; the morning glamour fades away,
And now before thy noontide self confessed
Stand all the joys withholden from thy youth;
And, certes, if thy soul dare speak the truth,
Thou wilt bow down in thankfulness, and say,
"They were unworthy, truly God knew best."

